# Low Grade Islet but Marked Exocrine Pancreas Inflammation in an Adult with Autoimmune Pre-Diabetes

**DOI:** 10.1155/2019/5863569

**Published:** 2019-12-19

**Authors:** Hiroshi Nomoto, Tatyana Gurlo, Madeline Rosenberger, Mark D. Girgis, Sarah Dry, Peter C. Butler

**Affiliations:** ^1^David Geffen School of Medicine at UCLA, Larry L. Hillblom Islet Research Center, Los Angeles, CA, USA; ^2^David Geffen School of Medicine at UCLA, Department of Surgery, Los Angeles, CA, USA; ^3^David Geffen School of Medicine at UCLA, Department of Pathology, Los Angeles, CA, USA

## Abstract

There is unexplained deficit in size and function of the exocrine pancreas in type 1 diabetes (T1D). We obtained pancreas from an individual with pre-T1D obtained at surgery and addressed the question, what is the relative inflammation in the exocrine and endocrine pancreas in pre-T1D in the absence of the potential confounding changes at autopsy or in brain dead organ donors. Pancreas was removed surgically from a 36 year woman for benign neuroendocrine tumors (NET). The patient had gestational diabetes at age 29 and has a 23 year old sister with T1D. Pre-operative fasting glucose of 109 mg/dl and HbA1C 5.8% revealed prediabetes with an anti-GAD 1,144 (5–250 U/ml) together with family history implying pre-T1D. There was patchy low grade immune infiltration in some, but not all, islets that met criteria for autoimmune insulitis. The exocrine pancreas showed more abundant inflammation with areas of chronic pancreatitis and acinar to ductal metaplasia, and with other areas of atrophy and fatty infiltration. In pre-T1D inflammation may be more prominent in the exocrine than the endocrine pancreas, calling into question the sequence of events and assumed islet centric basis of autoimmunity leading to T1D.

## 1. Introduction

Type 1 diabetes (T1D) is an autoimmune disease with an eventual near complete loss of beta cells [1]. In adult onset T1D (in those with slow progressing beta cell dysfunction sometimes referred to as latent autoimmune disease in adults, or LADA and by others as slowly progressive insulin dependent diabetes mellitus, or SPIDDM) [2, 3] there may be as much as a 5–10 year prediabetes phase following the detection of autoantibodies and clinical diabetes onset. Considerable effort has been expended on arresting disease progression during the prediabetes phase of T1D, but with limited success. This is partly due to a limited understanding of the sequence of events leading to loss of beta cell function in T1D. Recent studies of the endocrine pancreas of individuals with pre-T1D reveal a surprisingly low grade inflammatory infiltrate [4]. It is possible that this is due to the high dose glucocorticoid therapy typically administered to reduce cerebral edema prior to organ retrieval from brain dead organ donors. Also, brain death is known to be a potential confounder of immunopathology [5, 6]. The present reported case provided the rare opportunity to examine pancreas obtained at surgery from an ambulatory patient with pre-T1D in the absence of massive glucocorticoid therapy or effects of brain death.

It has also long been appreciated that there is an unexplained decrease in both size and function of the exocrine pancreas in T1D [3, 7, 8] with abnormalities in the ductal anatomy [9]. The small pancreatic size has recently been found to be already present in individuals with pre-T1D [10, 11]. The exocrine pancreas in both slowly progressive and fulminant forms of T1D have increased inflammatory cells that are comparable in subtypes to those observed in islets in the same individuals [12–14] as well as activation of the pancreatic duct compartment (PDG) [15], a regenerative compartment that is activated by pancreatitis. Taken together these studies have raised the possibility that inflammation originating in the exocrine pancreas may play a role in the pathophysiology of insulitis. It was therefore also of particular interest in the present case where surgically resected pancreas became available from an individual with pre-T1D to evaluate the exocrine and endocrine pancreas in the pre-T1D phase.

## 2. Case Presentation

A 36 year old woman presented to the UCLA medical center with abdominal pain. Previously at age 27 bilateral pheochromocytoma were resected surgically with biochemical and clinical resolution of catecholamine excess. There was no family history of endocrine neoplasia, and she was screened negatively for MEN1 and RET mutations associated with multiple endocrine neoplasia. The blood glucose on the day of surgery ranged from 95 to 126 mg/dl, and on discharge 5 days later fasting blood glucose was 104 mg/dl on no glucose lowering therapy. At age 29, the patient developed gestational diabetes. Diabetes resolved on delivery of a healthy baby girl. The patient's 23 year old sister has T1D treated with insulin. On examination she was well nourished (BMI 27 kg/m^2^). There was no clinical or biochemical evidence of recurrence of pheochromocytoma but a CT scan of the abdomen revealed 4 hypervascular well demarcated nodular lesions in the head and uncinate of the pancreas with marked fatty replacement of the pancreatic body and tail. The pancreas volume was approximately 50% of normal for age and gender. Cytological evaluation of an ERCP fine needle aspirant of a mass in head of pancreas was consistent with a neuroendocrine tumor. The hemoglobin 13.1 g/dL (11.6–15.2 g/dL), red cell count 4.22 × 10^6^/*µ*L (3.97–5.09 × 10^6^/*µ*L) and white blood cell count 5.05 × 10^3^/*µ*L (4.16–9.95 × 10^3^/*µ*L) were normal. Fasting blood glucose was 109 mg/dl increasing to 181 mg/dl with simultaneous plasma C-peptide 7.6 ng/ml 2 hours after a 75 g glucose tolerance test. HbA1C was 5.8%. Plasma lipase, liver function tests and renal function were normal. Anti-GAD was strongly positive 1,144 (5–250 U/ml), but autoantibodies to ICA-512, IA-2 and Zn8 (all evaluated more than two months preoperatively) were not detected. The HLA type of the patient was DRB1∗ 07:01:01, DRB1∗ 16:01:01, DRB4∗ 01:03:01, DRB5∗ 02:02, DQB1∗ 02:02:01, DQA1∗ 01:02, DQA1∗ 02:01:01.

A Whipple resection contained mass 2 × 4 cm in size that was composed of multiple neuroendocrine tumors. The mass had a tan-white semi-firm cut surface. The mass was less than 0.1 cm from the uncinate margin. The pancreatic duct 0.2 cm in diameter was identified within the Whipple resection and was clear of the tumor. Histologically the mass was a well differentiated neuroendocrine tumor (Supplementary [Supplementary-material supplementary-material-1]) with a Ki-67 labeling index of <1%. All margins of the neuroendocrine tumor tissue were clear with no necrosis, lymphatic or neural invasion identified. 25 regional lymph nodes were examined and none contained tumor. The tumor stained strongly positively for synaptophysin, weakly for chromogranin, negatively for insulin, glucagon, pancreatic polypeptide, somatostatin and glutamate decarboxylase (Supplementary [Supplementary-material supplementary-material-1]). Residual tail of pancreas was noted at surgery to be small and firmly adherent to surrounding structures consistent with the CT scan evaluation of the pancreas. The patient made an uneventful recovery post operatively. Diabetes was managed with 3–5 units of insulin glargine QHS with fasting glucose values in the 90–105 mg/dl range and corresponding C-peptide values of 0.7 and 2.5 ng/ml. One year later diabetes remains well controlled with insulin glargine 5 units.

**Figure 1:**
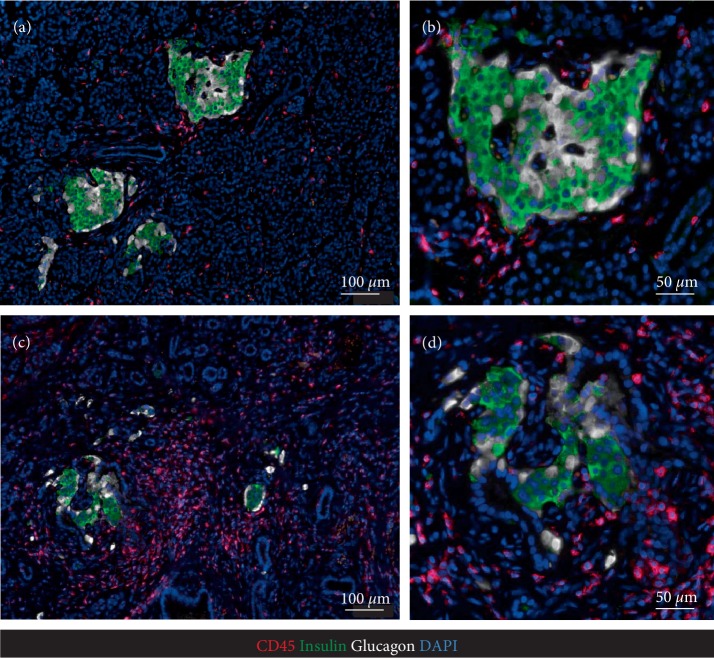
Representative immunofluorescence images of a pancreatic section stained for leucocyte common antigen CD45 (red), C-peptide (green), glucagon (white), nuclei DAPI (blue), illustrating variable immune infiltration of pancreatic parenchyma and islets. (b) and (d) are high magnification images of islets in (a) and (c) respectively. (b) shows the focal aggregation of CD45+ cells at one pole of the islet typical in early T1D and referred to as peri-insulitis.

**Figure 2:**
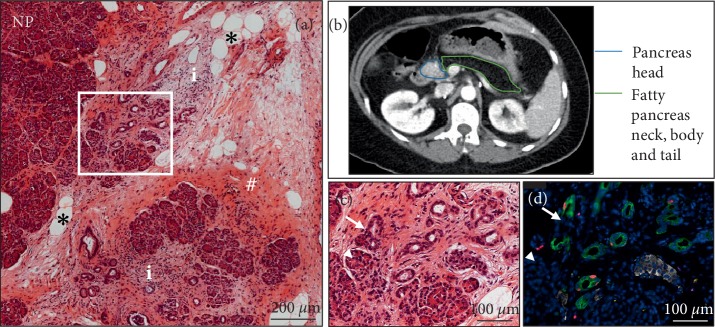
(a) Representative image of pancreatic section stained with H&E: NP-normal parenchyma, ^∗^—fat deposition, # — fibrosis, i —a site of inflammation next to the area of acinar-to-ductal metaplasia (box). (b) Cross-sectional view of CT scan slice of the pancreas showing normal density of the head of the pancreas while the neck, body and tail show low density consistent with fatty replacement acinar tissue. (c) High magnification of the boxed area (in (a)) illustrating acinar to ductal metaplasia (arrow) versus normal acinar tissue (arrow head). (d) Image of the same area in the adjacent section immunostained with anti-cytokeratin (green), Ki67 (red) and C-peptide (white).

**Figure 3:**
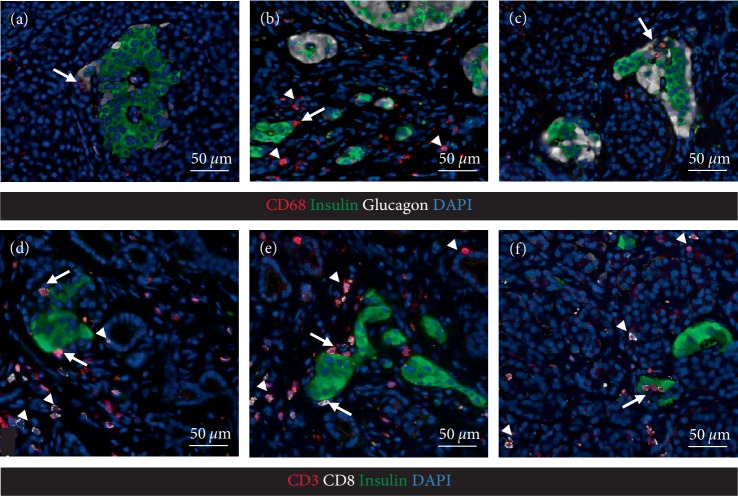
Representative immunofluorescence images of the pancreatic sections stained for macrophage marker CD68 (red), insulin (green), glucagon (white) in panels (a–c), or for T cell markers CD3 and CD8 (red and white respectively) and insulin (green) in panels (d and e); blue -nuclei (DAPI). Macrophages were found in peri-islet area (a–c, arrows) or pancreatic parenchyma (b, arrow head). CD8+CD3+ T cells were frequent throughout the parenchyma (d and e, arrow heads), in the areas of acinar-to-ductal metaplasia (d, e, arrow heads), and in the peri-islet area (d–f, arrows).

**Figure 4:**
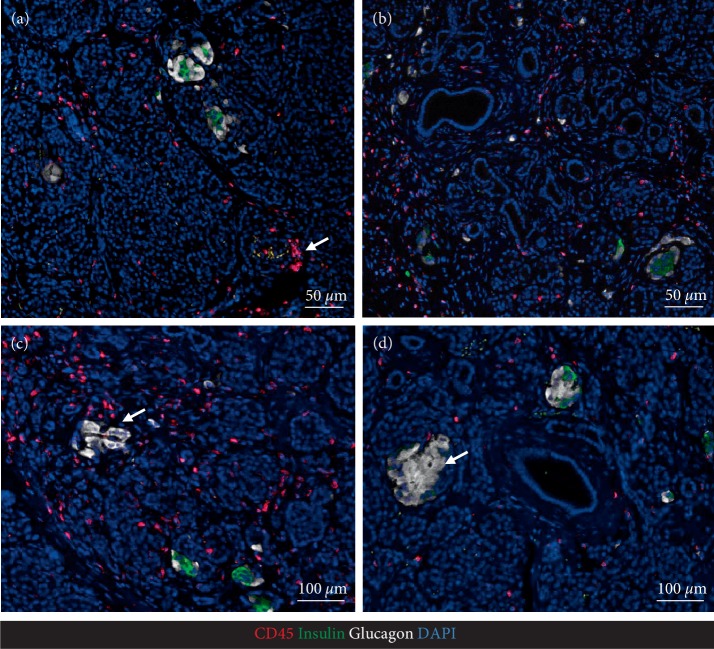
Representative immunofluorescence images of a pancreatic section stained for leucocyte common antigen CD45 (red), insulin (green), glucagon (white), nuclei DAPI (blue). Leucocytes are presented scattered throughout the exocrine pancreas as well as in occasional focal aggregates (a, arrow). Leucocytes are also present in areas of acinar-to-ductal metaplasia illustrated in (b). While most islets in this case had beta cells present, as expected in evolving type 1 diabetes, occasional islets were present with no detectable beta cells (c, arrow) or a few beta cells with minimally insulin immunoreactivity (d, arrow).

Having obtained IRB approval (IRB#17-001712) resected pancreas remote from the NETs was examined histologically as previously described [16, 17]. Tumor free pancreas (Figures 1 and 2) showed relatively normal islets with a pancreatic beta cell fractional area measured in sections stained for C-peptide being 0.57 ± 0.09%. There was a low grade and immune cell islet infiltrate that varied between islets that had no immune infiltrate to others that occasionally met the criteria of insulitis [18]. The mean immune infiltrate (illustrated in Figures 1 and 3) was 1.95 ± 0.21 CD45^+^, 1.47 ± 0.18 CD3^+^, 1.14 ± 0.14 CD8^+^, and 0.04 ± 0.00 CD68^+^ cells/islet section, with 3.4% of islets being negative for insulin (pseudo-atrophic islets, Figure 4(c)). Beta cell replication by Ki67 was not detected but areas of increased cell replication were present in areas of acinar to ductal metaplasia (Figure 2(d)). Beta cell apoptosis was not detected but increased TUNEL staining in areas of exocrine immune infiltration served as a positive control.

The exocrine pancreas showed more abnormalities than the endocrine pancreas with regions of chronic pancreatitis characterized by acinar to ductal metaplasia (Figures 2, 4(b) and 4(d), 4(e)), inflammation and fibrosis with areas of exocrine atrophy (Figure 2). These regions of inflammation were lobular in distribution as previously reported for recent onset T1D in humans and with no evidence of local ductal dilatation. CD45+ and CD3+ cells were frequently detected within exocrine pancreas parenchyma either as scattered single cells or as focal dense infiltrates (Figures 1(c) and 4(a)), and often associated with areas of acinar-to-ductal metaplasia (Figures 4(b) and 3(d), 3(e)). In relation to islets, CD45+ and CD3+ cells were mostly present in the islet periphery (peri-insulitis). Macrophages were less frequent in pancreatic parenchyma (Figure 3(b)), but were occasionally detected in the islet periphery (Figures 3(a) and 3(b)) and as a cluster inside islets (Figure 3(c)).

## 3. Discussion

The present case provided the opportunity to evaluate pancreas procured at surgery from an adult individual with pre-T1D (also potentially referred to as pre-LADA or pre-SPIDDM). It remains unresolved whether there is a distinct type of autoimmune mediated diabetes termed LADA originally described to distinguish adults that develop autoimmune mediated diabetes from type 2 diabetes when it was not widely recognized that T1D may occur at any age of life [2], as occurs in other autoimmune endocrinopathies such as Hashimoto's thyroiditis. The present case does not have a high risk HLA type for T1D [19], a finding that used to be considered a characteristic of LADA, but was subsequently challenged [20].

The point of particular interest in this case is that, to our knowledge, uniquely we had access to freshly procured pancreas from an individual with autoimmune pre-diabetes without the potential confounding effects of brain death, the ICU environment and massive corticosteroid therapy typically administered prior to retrieval of organs from brain dead organ donors. The latter might attenuate the inflammatory infiltrate in an autoimmune setting. Brain death has been reported to induce changes that impact islet vasculature and function, potentially introducing confounding effects on exocrine or endocrine pancreas through massive release of cytokines [5, 6]. We should mention that the pancreatic neuroendocrine tumors in this case also present a potential confounding influence on the exocrine pathology. While the tumor cluster did not involve the main pancreatic duct, and lipase levels were consistently normal, it is plausible that some of the exocrine changes might be a consequence of the tumors rather than autoimmunity. Also, while the patient screened negatively for the known genetic variants for multiple endocrine neoplasia, the fact that she previously had biliateral pheochromocytomas before developing a complex of neuroendocrine tumors in the pancreas implies she may have an as yet undescribed genetic predisposition to endocrine tumors. Anti-GAD antibodies have been reported in individuals with GAD expressing neuroendocrine tumors and stiff man syndrome [21]. The tumor in the present patient did not express GAD and the patient had no features consistent with stiff man syndrome.

The patient had a fasting blood glucose and HbA1C in the prediabetes range but marked glucose intolerance. Uniquely in the present patient, we were able to document that the impaired fasting glucose and glucose intolerance was already present despite minimal beta cell loss and low grade islet inflammation. Prospective clinical trials have noted a progressive decline in glucose tolerance in the pre-T1D phase [22], characterized by both a progressive defect in insulin secretion [23, 24] and increasing insulin resistance characteristic of T1D, plausibly due to declining pulsatility of insulin secretion [25, 26].

The present case also provided a unique opportunity to evaluate the exocrine pancreas in pre-T1D. Radiological findings and subsequent direct observation at surgery revealed a relatively atrophic pancreas of ~50% volume expected for age with fatty infiltration in the tail. There is exocrine pancreas atrophy with increased pancreatic fat with advanced age [17] but this does not impact the endocrine pancreas that remains well preserved. Increased pancreatic fat in type 2 diabetes is attributable to associated obesity, but since pancreatic fat is comparable in obese individuals with and without type 2 diabetes [27], pancreatic fat does not appear sufficient to cause diabetes. Therefore the accumulation of fat and exocrine atrophy do not seem likely mediators of beta cell failure in the presented case. There was lobular low grade chronic pancreatitis confirmed histologically. While it is possible that the areas of exocrine pancreas immediately adjacent to the NETs in the head of the pancreas might be impacted by the tumors, it was notable that by palpation the tail of the pancreas was firm, atrophic and adherent to surrounding structures consistent with chronic low grade pancreatitis. Also, plasma lipase levels were normal on multiple occasions that would be unusual for obstructive pancreatitis and that there was no discernable pancreatic duct dilatation macroscopically or histologically as would be expected with obstructive pancreatitis.

Finally, the exocrine pancreatic findings in this case mirror those reported in pre-T1D from brain dead organ donors [10] in the absence of tumors.

## 4. Conclusion

In conclusion, in the present case of apparent pre-T1D, the low level of insulitis reported in autoantibody positive brain dead organ donors with and without T1D is reproduced, assuring that this finding is not an artifact consequent on high doses glucocorticoids given prior to organ procurement. Also, the finding of prominent exocrine changes of immune inflammation, acinar to ductal metaplasia and acinar atrophy in the pre-T1D phase are supportive of studies that implied that exocrine inflammation may contribute to development of islet inflammation eventually leading to T1D [3, 9, 12–14]. The present case supports those findings of low grade islet immune cell infiltration but more prominent changes in exocrine pancreas extended here to pre-T1D consistent with loss of pancreas volume in pre-T1D [11]. As yet the origin of the exocrine inflammation in T1D and its role in the eventual loss of beta cell function and mass remains unknown.

## Abbreviations

NET: Neuroendocrine tumor
T1D: Type 1 diabetes
Anti-GAD: Antibody against glutamic acid decarboxylase
PDG: Pancreatic duct gland
LADA: Latent autoimmune disease in adults.

## Supplementary Materials

Supplementary Materials Supplementary Figure 1: no insulin, glucagon, pancreatic polypeptide, somatostatin or glutamate decarboxylase (GAD) was detected in the tumor. (A) Representative bright field image of resected tumor with the margin of the pancreas parenchyma containing islets of Langerhans stained for insulin (brown). Dotted line identifies the tumor margin. (B) Representative immunofluorescence image of the area shown in panel A on the adjacent section stained for glucagon (white), pancreatic polypeptide (red) and somatostatin (green); blue — nuclei (DAPI). (C) Ki67 staining in tumor (brown, arrow). (D) Representative immunofluorescence image of the tumor section stained for synaptophysin (green) and chromogranin A (CHGA, red).
