# Supplementary material for: Low Grade Islet but Marked Exocrine Pancreas Inflammation in an Adult with Autoimmune Pre-Diabetes
Source: Case Rep Endocrinol. 2019 Dec 19;2019:5863569. doi: 10.1155/2019/5863569 (PMC6948281; doi:10.1155/2019/5863569)

**Supplementary Figure 1.** No insulin, glucagon, pancreatic polypeptide, somatostatin or glutamate decarboxylase (GAD) was detected in the tumor. (A) Representative bright field image of resected tumor with the margin of the pancreas parenchyma containing islets of Langerhans stained for insulin (brown). Dotted line identifies the tumor margin. (B) Representative immunofluorescence image of the area shown in panel A on the adjacent section stained for glucagon (white), pancreatic polypeptide (red) and somatostatin (green); blue – nuclei (DAPI). (C) Ki67 staining in tumor (brown, arrow). (D) Representative immunofluorescence image of the tumor section stained for synaptophysin (green) and chromogranin A (CHGA, red).


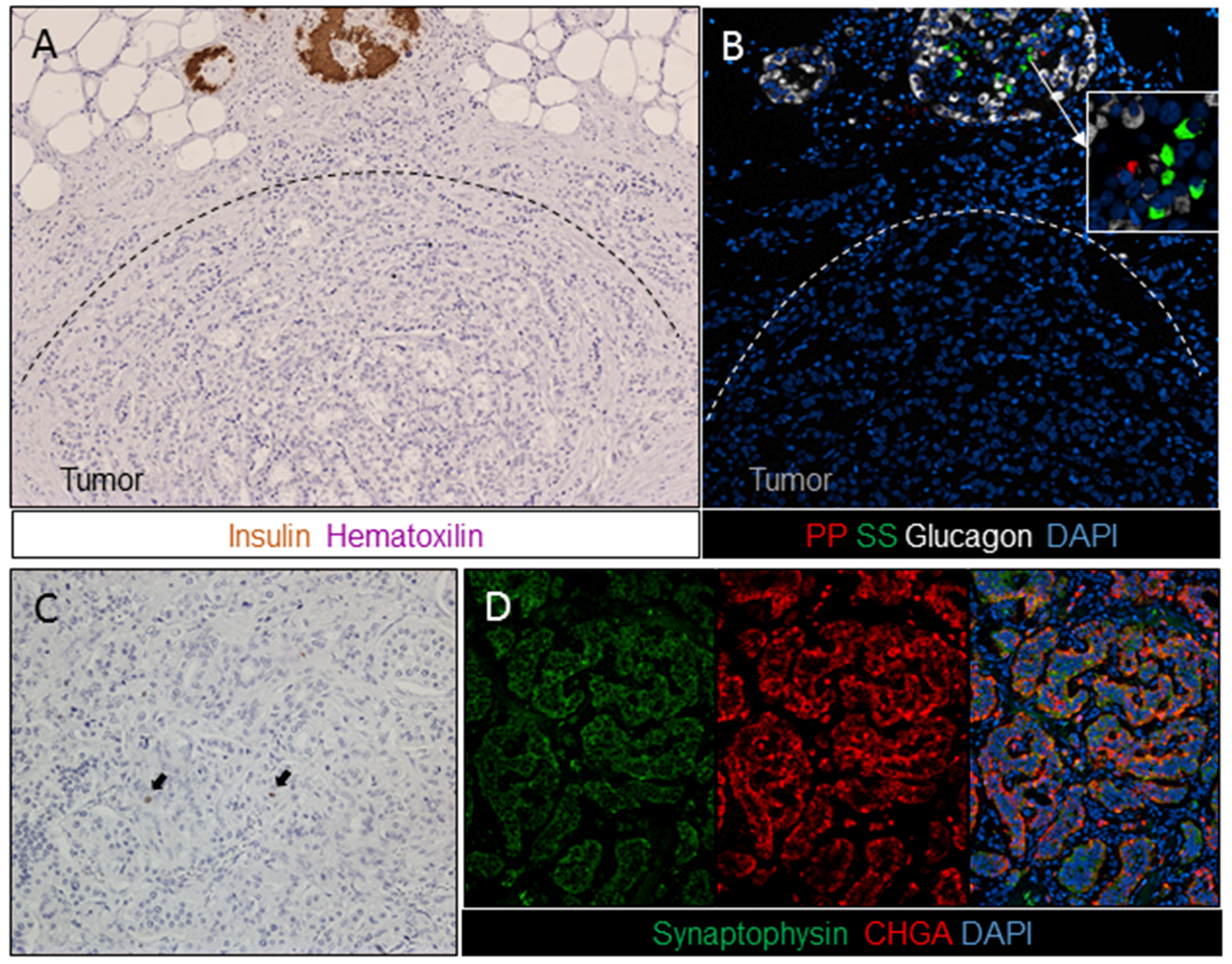

Supplement: Supplementary Materials — Supplementary Figure 1: no insulin, glucagon, pancreatic polypeptide, somatostatin or glutamate decarboxylase (GAD) was detected in the tumor. (A) Representative bright field image of resected tumor with the margin of the pancreas parenchyma containing islets of Langerhans stained for insulin (brown). Dotted line identifies the tumor margin. (B) Representative immunofluorescence image of the area shown in panel A on the adjacent section stained for glucagon (white), pancreatic polypeptide (red) and somatostatin (green); blue — nuclei (DAPI). (C) Ki67 staining in tumor (brown, arrow). (D) Representative immunofluorescence image of the tumor section stained for synaptophysin (green) and chromogranin A (CHGA, red). [file 5863569.f1.docx]
